# When Teamwork Works: Examining the Relationship Between Leader-Member Exchange Differentiation and Team Creativity

**DOI:** 10.3389/fpsyg.2021.646514

**Published:** 2022-01-21

**Authors:** Juan Du, Xinyue Lin, Yahua Cai, Fufu Sun, Joseph Amankwah-Amoah

**Affiliations:** ^1^School of Business Management, Shanghai International Studies University, Shanghai, China; ^2^Department of Human Resource Management, Shanghai University of Finance and Economics, Shanghai, China; ^3^Kent Business School, University of Kent, Chatham, United Kingdom

**Keywords:** team creativity, team behavioral integration, LMX differentiation, team emotional intelligence, social comparison theory

## Abstract

Drawing on team creativity literature and social comparison theory, we investigate how leader-member exchange (LMX) differentiation influences team creativity. Using a survey based on 91 R&D teams from Chinese companies, we observe that LMX differentiation is negatively related to team creativity (β = −0.35, *p* < 0.01). More importantly, we demonstrate that team behavioral integration mediates the relationship between LMX differentiation and team creativity (indirect effect size = −0.72, with 95% CI of −1.91, −0.13), and team emotional intelligence (TEI) moderates the relationship between LMX differentiation and team behavioral integration (β = 0.23, *p* < 0.05), such that LMX differentiation has a weaker negative influence on team behavioral integration when TEI is higher. These results provide relevant suggestions for organizational team building, management, and development.

## Introduction

In an increasingly changing and competitive work environment, creativity has become an essential ingredient for organizations’ survival and development (Anderson et al., 2014). Defined as the product of novel and useful ideas by a group of employees working together (Shin and Zhou, 2007), team creativity has garnered a growing body of research in innovation and strategy literature. Accordingly, an increasing number of organizations are adopting teams as the primary work units to maximize creative processes (Li et al., 2010).

Although a number of researchers have explored the impact of leadership influence on team creativity (Shin and Zhou, 2007; Zhang et al., 2011; Hu et al., 2017; Mo et al., 2019), there are notable shortcomings in the current literature. First, despite the growth in literature, the potential influence of leader-member exchange (LMX) differentiation on team creativity has not been given as much attention. Among the limited present studies that have examined the direct effect of LMX differentiation on team creativity, there remains a major inconsistency in the current findings (Li et al., 2016; Matta and Van Dyne, 2020). As demonstrated by a review by Anand et al. (2015, p. 288), the “findings on the effects of LMX differentiation have been mixed at best.” Indeed, the current literature lacks insights on the mechanisms that drive differential effects (Matta and Van Dyne, 2020).

As one of the most fruitful fields in leadership research during the past few decades, LMX theory captures the notion that different relationships with leaders significantly impact employees’ performance (Gerstner and Day, 1997). LMX differentiation, one major component of LMX theory, is defined as the degree to which members working with the same leader differ in terms of their relationship quality with their leaders (Ma and Qu, 2010). This varying exchange relationship quality then promotes or mitigates subordinates’ performance including their self-views and employee’s voice behavior (Martin et al., 2016; Matta and Van Dyne, 2020). Although some studies support LMX differentiation as having an influence on team processes and outcomes (Harris et al., 2014; Liao et al., 2017; Matta and Van Dyne, 2020), the effect of LMX differentiation on team creativity remains underexplored. With this in mind, the primary goal of this study is to examine the effects of LMX differentiation on team creativity. Social comparison theory points out that people make self-attribution comparisons both consciously and unconsciously (Festinger, 1954). The equality principle of fairness is one of the main principles within social comparison theory. High level of LMX differentiation makes team members comparison more salient, that is, team members are easy to perceive the differential treatment from leaders particularly under team context (Liden et al., 2006). Since the development of team creativity requires cooperation and information sharing among the team members, the presence of high LMX differentiation would lead to the perception of inequality, which is thought to destroy the harmony and cooperation within work teams (Liao et al., 2017), thus affecting team creativity (Camps et al., 2019; Graso et al., 2020).

The study on the context of teamwork is critical to explore how team-level constructs and their interactions influence team outcomes. We postulate that team emotional intelligence (TEI) will moderate the effect of LMX differentiation on team behavioral integration. TEI is the degree of the emotional intelligence that team members appear to use when they interact with each other. High TEI suggests that team members can better regulate their emotions and can better prioritize organizational issues (Salovey and Mayer, 1990). In this condition, team members can pay less attention to the effects of LMX differentiation and instead focus on the things that benefit the greater team(s), which can attenuate the team conflict whereby caused by LMX differentiation. Taken together, we postulate that TEI can alleviate the negative effect of LMX differentiation on team behavioral integration.

This study offers several vital contributions to the existing literature. First, building upon the existing literature on team performance research (Sui et al., 2016), we provide a more comprehensive understanding of LMX differentiation and its effect on team creativity. In this direction, we validate the effect of LMX differentiation on upper-level team creativity, which is still in its infancy stages of development (Li et al., 2016; Qu et al., 2017). We also shed new lights on the influence of LMX differentiation on team behavioral integration and help to explain why and how the team climate with high level of LMX differentiation decreases team behavioral integration. In addition, by exploring team behavioral integration as the mediator, we extend our empirical understanding of the outcomes of LMX differentiation and provide an in-depth explanation of the team member interaction process in this relationship within general teams. Last, we infer that TEI may interact with LMX differentiation to then affect team behavioral integration. This provides a new perspective to better understand how emotional intelligence works at the team level, by demonstrating when these effects occur.

The rest of the manuscript is organized along the following lines. In the subsequent section, a review of LMX differentiation and team creativity is presented. We then present the research method and analysis of the results. The final section outlines the different theoretical and practical implications of the study.

## Theoretical Background and Hypotheses Development

According to the input-process-outcome model (IPO, McGrath, 1984), LMX differentiation is the element of “input” and team creativity is element of the “output,” and the “process” describes how “input” is transformed into “output,” including social exchange and interaction [e.g., communication, cooperation, and information sharing (Marks et al., 2000)]. Team behavioral integration, which reflects the degree of convergence of team member interactions and demonstrates their collective behavior, is a critical construct capturing the social interaction among team members (Marks et al., 2000). Based on social comparison theory, LMX differentiation violates the social principle of equality and triggers team conflict, which could negatively affect team behavioral integration. Team behavioral integration includes team’s information exchange, collaborative behavior, and joint decision-making (Simsek et al., 2005), all of which have deep influences on team creativity. Thus, we propose that team behavioral integration may be a crucial intervening process that might explain the relationship between LMX differentiation and team creativity.

### Leader-Member Exchange Differentiation and Team Creativity

In recent three decades or so, many organizations have shifted from solely focusing on the individual worker or star performers to innovate to focus on cultivating and developing teams (Groysberg and Abrahams, 2006; Groysberg, 2010; Adomako et al., 2019; Amankwah-Amoah, 2020). Indeed, sole stars in organizations have been found to be a myth as individual performance is increasingly buttressed by colleagues and supporting cast (Groysberg and Abrahams, 2006; Groysberg, 2010). In the modern work environment, there are new assumptions for organizational creativity, in that creativity at work is usually conducted within team settings (van Knippenberg et al., 2011). Organizational scholars and practitioners alike have explored how to promote team creativity, a prominent indicator of team performance (Tu et al., 2019).

Team creativity is not the simple sum of individual creativity, but rather, it involves a complex team members’ interaction process and can be methodologically examined using self-report, social network analysis, focus groups, and mixed method designs (Akhtar et al., 2019). Existing studies have highlighted the importance of team members’ individual propensity for creativity in the team creativity process (Hülsheger et al., 2009; Kim et al., 2013). Boundary conditions of team creativity include the presence or absence of positive organizational culture, psychological safety, and team trust (Boon et al., 2016; Han et al., 2019).

Social comparison theory suggests that people make self-attributional comparisons both consciously and unconsciously and is a useful heuristic for how creativity is enacted and shared within teams (Festinger, 1954). If an individual feels as though their peers are empowered to enact creativity, they are more likely to exhibit creativity as a result (Amabile, 2018). In the organizational context, leaders, including those who enact transformational leadership behaviors, are largely the vehicles to which employees feel empowered to enact goal-directed behaviors, which also include creativity (Dong et al., 2017). Thus, if a leader is treating their subordinates differently (i.e., when LMX differentiation is high), employees are likely to become disengaged and less inclined to initiate collaborative work behavior (Roter, 2017).

Leader-member exchange differentiation suggests that within-group variability of the quality of the leader-follower relationship is different among certain employees (Liden et al., 2006; see also Dong et al., 2020). The different treatment makes team members comparison more salient and the work environment more competitive, suggesting that team members are easy to perceive the differential treatment from leaders particularly if they work together every day. This comparison can significantly influence work outcomes. Specifically, if the level of LMX differentiation within a team is high, then the perceived unfairness between team members will be likely experienced. This in turn may cause potential conflict and less cooperation, thereby harming team creativity (Li and Liao, 2014; Hopkins and Yonker, 2015). Additionally, team members may have better relationships and more interpersonal interactions with those who have similar LMX differentiation quality, and alienating those whose LMX quality is significantly different from their own (Brewer, 1999). Therefore, high LMX differentiation may lead to differences in in-group and outgroup perception. Team members in similar high- and low-quality LMX relationships will likely form coalitions, which will lead to increased interpersonal and emotional conflicts across these teams (Hooper and Martin, 2008). This process will exert negative impacts on team members’ social interactions, thus mitigating team creativity.

In addition, the equality principle of fairness within social comparison theory may directly explain the negative relationship of LMX differentiation on team creativity (Camps et al., 2019; Graso et al., 2020). The development of team creativity requires cooperation and information sharing among the team members. However, the presence of high LMX differentiation would lead to the perception of inequality, which is thought to destroy the harmony and cooperation within work teams (Liao et al., 2017). Altogether, perceived inequity in the context of social comparison is harmful to team creativity. Thus, we hypothesize the following:

*H1: LMX differentiation is negatively related to team creativity.*

### The Mediating Role of Team Behavioral Integration

The concept of “behavioral integration” was first put forth by Hambrick and Mason (1984) within the framework of Upper Echelon theory. This theory reflects the degree of convergence of team member interactions and demonstrates their collective behavior. There are three dimensions of behavioral integration: quality and quantity of team information exchange, cooperation behavior, and collective decisions. Each dimension reinforces and promotes the others and explains how a team operates and works together (Hambrick and Mason, 1984). Team behavioral integration has been linked to be a crucial process factor with great benefits for team outcomes (Bingyan et al., 2016; Tekleab et al., 2016).

We posit that LMX differentiation may have a negative effect on team behavioral integration. First, as aforementioned, LMX differentiation leads to the perception of relational boundaries in teams, which in turn makes team members form in-group and outgroup norms (Brewer, 1999; Anand et al., 2011). In-group members possess more valuable team resources, such as key positions, attention from others, and are likely to garner more promotions through the high-quality relationships with leaders (Weeks et al., 2017). This situation makes outgroup members feel higher levels of perceived unfairness and lower levels of organizational justice perceptions, therefore undermining team behavioral integration by increasing conflict (Lim and Loosemore, 2017). High LMX differentiation destroys team members’ justice perception (Liao et al., 2017). Perceived unfairness makes team members disappointed, frustrated, and angry, which reduces their efforts to enact teamwork and harms team members’ coordination (Hooper and Martin, 2008). In conclusion, LMX differentiation is harmful to acquire both the quality and quantity of team information exchange, cooperation behavior, and collective decisions. When teams’ behavioral integration is high, members working together invest more time and energy in identifying problems, searching for more information, and putting forward effort in knowledge creation (Kim, 2010). Teams with high levels of behavioral integration are characterized by having open and timely communication of information among team members, habitual teamwork, and joint decision-making (Sousa and Van Dierendonck, 2016; Tekleab et al., 2016). When team members effectively use behavioral integration, they can obtain more valuable information, knowledge, and ideas, which in turn improves engagement in the above behavioral processes related to creativity. Moreover, a team with higher behavioral integration enables team members to collaborate with different people and experience more diverse ways of thinking, which then enriches and expands members’ thinking patterns, which also contributes to team creativity (Hoever et al., 2012). Accordingly, we hypothesize the following:

*H2: Team behavioral integration mediates the relationship between LMX differentiation and team creativity.*

### The Moderating Role of Team Emotional Intelligence

Team emotional intelligence is the capacity to understand and effectively manage our emotions, while attending to the social emotions of others (Sy et al., 2006; Mayer et al., 2016). TEI has four dimensions: awareness of own emotions, awareness of others’ emotions, management of own emotions, and management of others’ emotions (Jordan and Lawrence, 2009). The interaction of individual traits and complex situational factors, such as team size, industry, and job function, makes the teamwork outcome not the same as the sum of individual self-report data (Li et al., 2010). Accordingly, TEI is reflectively not the sum of individual emotional intelligence within a team, but rather, it is how team members appear to use their individual emotional intelligence when they interact with each other (Jamshed and Majeed, 2019; Lee and Wong, 2019).

In this study, we propose that TEI may weaken the negative effects of high LMX differentiation on team behavioral integration. First, teams with high levels of emotional intelligence are better at perceiving others’ emotions and understanding others’ attitudes, goals, and behavioral intentions more accurately (Van Kleef et al., 2009). Employees with high emotional intelligence both high and low LMX quality are better able to adjust their negative emotions that resulted from LMX differentiation. Team members with high emotional intelligence and high LMX quality can easily capture the negative emotions of low LMX quality coworkers provide them timely care, and help them to regulate their emotions. Members besides, members with high TEI have a greater propensity to focus their attention on task-relevant issues (Salovey and Mayer, 1990; Sui et al., 2016; Martin et al., 2018). Although LMX differentiation damages the fairness and may trigger relational boundary between in-group and outgroup members, both of them pay their much attention to accomplish team goals, so team members could illustrate their viewpoint, exchange information, and cooperate with each other effectively. Second, previous literature has indicated that TEI plays an important role in team members’ behavioral interaction (Hopkins and Yonker, 2015), teams with low emotional intelligence which lacking of the ability to manage emotions experience more task conflict and relationship conflict (Ayoko et al., 2008), whereas teams with higher emotional intelligence have less task conflict and relationship conflict that increases the team behavioral integration (Yang and Mossholder, 2004). Jordan and Troth (2004) also examined that teams with higher levels of emotional intelligence are more likely to use an integrative conflict management style in a teamwork which focuses on the awareness of management of perceived and felt emotions both on themselves and others. TEI is an important process in which team members can adaptively shape and behave differently according to shifting environments, including the specific situation that is occurring within a team (Roberts et al., 2001). It is the process that highlights the nature of “intelligence” which is defined by Roberts et al. (2001) as “adaptation to, selection of, and shaping of the real-world environments relevant to one’s life.” As Elfenbein (2006) notes, “therefore, TEI is often a matter of effective interpersonal behaviors rather than unchangeable traits” (Elfenbein, 2006, p. 178). Therefore, TEI becomes an important indicator of the relationship between leader-member exchange and the successful integration of team behavior.

*H3: TEI moderates the relationship between LMX differentiation and team behavioral integration, such that LMX differentiation has a weaker negative influence on team behavioral integration when TEI is higher.*

Finally, combining H2 and H3, we also posit that TEI not only moderates the impact of LMX differentiation on team behavior, but will also moderate the indirect effect of LMX differentiation and team creativity, *via* team behavioral integration. When teams possess high emotional intelligence, the effect of LMX differentiation on team behavioral integration, and ultimately on team creativity, will be weaker. Conversely, in teams with low TEI, the indirect effect of LMX differentiation on team creativity through team behavioral integration will be stronger.

*H4: TEI moderates the strength of the mediated relationships between LMX differentiation and team creativity via team behavioral integration, such that the mediated relationship will be weaker under high TEI than under low TEI.*

**FIGURE 1:**
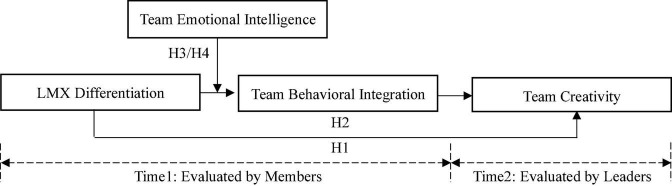
The theoretical model.

Based on the above, the research model was drawn as shown in Figure 1.

## Materials and Methods

### Participants and Procedure

In this study, we selected research and development (R&D) teams as our target subjects because this type of team requires higher levels of creativity, and employees communicate with leaders and colleagues frequently. We investigated R&D teams from eight diverse enterprises and research institutes involving machinery, electronic communication, high-speed railway, aerospace, software service, and other industries. Organizations were primarily located in Shanghai, Sichuang, and Hubei in China. We defined a team as a group of workers ranging from 3 to 10 members reporting to the same leader (Macht et al., 2019). After obtaining permission and support from relevant leaders of the surveyed enterprises, we randomly selected a total of 145 teams with each team consisting of one leader and 3–10 members.

To avoid common method variance (Podsakoff et al., 2003), data were gathered from different sources with the time lag of 1 month. At time point 1 (March 2018), employees filled out the subordinate questionnaire that included items measuring their perception of leader-member exchange and team behavioral integration, and their own level of emotional intelligence. At time point 2 (April 2018), leaders filled out the leader questionnaire that included items measuring entire teams’ creativity level, and team information, such as team size and task characteristics of teams (task complexity and task interdependence). Both team leaders and members self-reported their demographic information (age, gender, and education). Given that all surveys were administered during working hours, informed consent was obtained, all participants were not compensated for their involvement in the study, and all data were held confidential upon analysis.

We distributed a total of 640 questionnaires to employees at time point 1. A total of 483 surveys were returned with a response rate of 75.5%, with a final of 401 subordinate questionnaires obtained after eliminating the uncompleted and unmatched questionnaires, an effective completion rate of 62.7%. We distributed a total of 145 questionnaires to leaders at time point 2. A total of 108 surveys were returned with a response rate of 74.5%. At time point 2, a final of 91 leader questionnaires were obtained with an effective response rate of 62.8%.

Demographic characteristics of team members and team leaders were collected. Among team members, 57.8% were men and 42.2% were women; the main age groups were 26–35 years (26–30 accounting for 50.6%, 31–35 accounting for 26.8%); participants had relatively high educational levels (40.2% Bachelor’s degree, 36.4% Master’s degree). Among team leaders, 79% were men and 21% were women; the main age groups were above 36 years old (36–40 accounting for 21.5%, greater than or equal to 41 accounting for 65%); they have relatively high educational levels (31% Bachelor’s degree, 50% Master’s degree).

### Measures

We designed the questionnaire based on valid scales in the existing literature. The survey was initially constructed in English, and all items were translated into Chinese by conducting back-and-forth translation procedures (Brislin, 1986) to ensure the accuracy of translation. For most items, we adopted a six-point Likert scale, ranging from “1 – strongly disagree” to “6 – strongly agree.” Team creativity uses a five-point scale.

#### Leader-Member Exchange Differentiation

We used the 7-item scale developed by Wang et al. (2005) to measure LMX. A sample item is “my supervisor behaves in a manner thoughtful of my personal needs.” The McDonald’s ωfor the LMX scale is 0.91. Consistent with previous research, we aggregated the individual-level LMX scores into team-level LMX mean and measured LMX differentiation using the coefficient of variation (team LMX SD/LMX mean, Martin et al., 2018).

#### Team Behavioral Integration

We used the 4-item scale developed by Li and Hambrick (2005) to measure team behavioral integration. A sample item is “all team members have a voice in team decisions.” The McDonald’s ωfor the whole scale is 0.92. Given that team behavioral integration is a team-level construct but evaluated by individuals in this study, we aggregated these data into team-level ones by calculating the average value of team behavioral integration at the individual level in each team. We used rwg, ICC (1), and ICC (2) indicators to assess whether the measurement of this construct had sufficient intragroup consistency and intergroup heterogeneity (James et al., 1984; Bliese, 2000). The aggregation statistics were sufficient, with ICC (1) = 0.35, ICC (2) = 0.70, and mean rwg_(j)_ = 0.91.

#### Team Emotional Intelligence

We used Wong and Law’s Emotional Intelligence Scale (WLEIS) developed by Wong and Law (2002) to measure team members’ emotional intelligence. It contains 16 items to measure four subscales, with four items for each subscale: Self-Emotions Appraisal (SEA), Others-Emotions Appraisal (OEA), Use of Emotion (UOE), and Regulation of Emotion (ROE). We focus on team members’ whole emotional intelligence, and the McDonald’sωfor the whole scale is 0.93. The average rwg_(j)_ across subjected teams was 0.85. The ICC (1) value was 0.15, and the ICC (2) value was 0.52. We aggregated the individual-level emotional intelligence scores for each team to represent the respective team-level construct.

#### Team Creativity

We used the 4-item scale developed by Shin and Zhou (2007) to measure team creativity. It is a 5-point scale ranging from 1 (needs much improvement) to 5 (excellent). A sample item is “How creative do you consider this team to be?” The McDonald’s ω for this scale was 0.90.

#### Control Variables

In line with previous LMX and LMX differentiation research (e.g., Tse and Ashkanasy, 2015), we included team members’ demographic information (i.e., age, gender, and education), and also team size and task characteristics as control variables in the current research.

Additionally, we used the scale developed by Dean and Snell (1991) to measure task characteristics. The scale contains three items to measure task complexity (e.g., “to what extent do the jobs involve solving problems?”) and six items to measure task interdependence (e.g., “how much do people in this team have to coordinate work with others?”). The Cronbach’s α for task complexity and interdependence is 0.89 and 0.92, respectively.

## Results

### Confirmatory Factor Analysis

We conducted a series of confirmatory factor analysis with robust maximum likelihood estimator to explore the distinctiveness of the focus four variables at individual level. As shown in Table 1, the hypothesized four-factor model [*X*^2^/_(428)_ = 3.73 (<5), RMSEA = 0.05 (<0.05), SRMR = 0.04 (<0.05), CFI = 0.91 (>0.90), TLI = 0.92 (>0.90)] fitted the data better than alternative models, providing support for the distinctiveness of the four constructs in this study.

**TABLE 1:** Comparison of measurement models.

Models	*X* ^2^	d*f*	*X*^2^/d*f*	RMSEA	SRMR	CFI	TLI
Hypothesized four-factor model: LMX, TBI, TEI, and TC	1,596.44	428	3.73	0.05	0.04	0.91	0.92
Alternative three-factor model: LMX, TBI + TEI, and TC	2,284.3	431	5.30	0.13	0.09	0.72	0.68
Alternative two-factor model: LMX + TBI + TEI and TC	2,892.44	433	6.68	0.19	0.21	0.61	0.65
Alternative single-factor model: LMX + TBI + TEI + TC	4,626.44	434	10.66	0.23	0.21	0.66	0.54

*N = 401.*

*LMX, leader-member exchange; TBI, team behavioral integration; TEI, team emotional intelligence; TC, team creativity.*

*“ + “ represents two factors merged into one.*

### Descriptive Statistics and Correlations

Table 2 shows the means, standard deviations, correlations, and reliability coefficients of the variables.

**TABLE 2:** Means, standard deviations, and correlations among variables.

Variables	*M*	SD	1	2	3	4	5	6	7	8	9	10
(1) Team Size	4.38	1.02										
(2) Age	2.37	0.73	0.04									
(3) Gender	1.59	0.25	−0.10	0.16								
(4) Education	3.89	0.90	0.19	0.24*	−0.06							
(5) Task complexity	4.53	0.63	−0.15	0.21*	0.21*	0.03						
(6) Task interdependence	3.81	0.89	0.05	0.27**	−0.09	−0.10	0.45**					
(7) LMXD	0.08	0.05	−0.35**	–0.09	0.08	−0.13	−0.03	−0.02	/			
(8) TBI	4.47	0.69	0.05	–0.16	−0.06	−0.14	0.01	0.08	−0.21*	(0.92)		
(9) EI	4.38	0.30	0.12	−0.34**	−0.04	−0.15	−0.03	0.20	0.09	0.46**	(0.93)	
(10) TC	3.40	0.52	−0.04	0.23*	0.19	0.26*	0.23*	0.06	−0.32**	0.37**	0.12	(0.90)

*N = 91.*

*p < 0.05; **p < 0.01.

*Reliability estimates appear in parentheses across the diagonal. Task-c, task complexity; Task-d, task interdependence; LMXD, leader-member exchange differentiation; TBI, team behavioral integration; EI, emotional intelligence; TC, team creativity. Some control variables were coded as a dummy variable: gender (1 = female, 2 = male), age (1 = less than or equal to 25, 2 = 26–30, 3 = 31–35, 4 = 36–40, and 5 = greater than or equal to 41), education (1 = junior high school or below, 2 = high school, 3 = junior college, 4 = bachelor, 5 = master, and 6 = doctoral degree).*

### Hypotheses Testing

We used IBM SPSS 22.0 software to conduct hypotheses testing using ordinary least squares regression since our variables were the same level of analysis (team level). The results are shown in Table 3. The results in Table 3 indicate that LMX differentiation is negatively related to team creativity (M4, β = −0.35, *p* < 0.01). Hypothesis 1 was thus supported.

**TABLE 3:** Results of mediation and moderation analysis.

Variables	TBI			TC		
	M1	M2	M3	M4	M5	M6
**CV**						
Team size	−0.02	−0.09	−0.06	−0.17	−0.08	−0.16
Age	−0.18	0.03	0.05	0.09	0.19	0.16
Gender	−0.01	−0.05	−0.05	0.17	0.16	0.18
Education	−0.12	−0.10	−0.12	0.24	0.29**	0.28
Task-c	−0.00	0.04	0.04	0.12	0.15	0.12
Task-d	0.11	−0.07	−0.07	0.02	−0.05	−0.02
**IV**						
LMXD	−0.24*	−0.29**	−0.27**	−0.35**		−0.26**
**Mediator**						
TBI					0.45***	0.40***
**Moderator**						
TEI		0.50***	0.42***			
**Interaction**						
LMXD × TEI			0.23*			
*F*	1.34*	4.22***	4.52***	4.20**	6.39***	6.92***
*R* ^2^	0.10	0.29	0.33	0.26	0.35	0.40
Adjusted *R*^2^	0.03	0.22	0.26	0.20	0.30	0.35
Δ*R*^2^	/	0.19	0.04	/	/	0.15

*N (team) = 91.*

*p < 0.05; **p < 0.01; ***p < 0.001.

*All control variables were aggregated (the average value of each team). Task-c, task complexity; Task-d, task interdependence; LMXD, leader-member exchange differentiation; TBI, team behavioral integration; TEI, team emotional intelligence; TC, team creativity.*

We then examined the mediating effect of team behavioral integration between LMX differentiation and team creativity, following Baron and Kenny’s (1986) recommended four conditions for establishing mediation. LMX differentiation was negatively related to team behavioral integration (M1, β = −0.24, *p* < 0.05); LMX differentiation was negatively related to team creativity (M4, β = −0.35, *p* < 0.01); team behavioral integration was positively related to team creativity (M5, β = 0.45, *p* < 0.00); when team behavioral integration was added, the relationship between LMX differentiation and team creativity was weaker, albeit still significant (M6, β = −0.26, *p* < 0.01), which suggests partial mediation. To further assess the significance of the mediation, we applied the Model 4 of PROCESS (Hayes, 2012) to test the indirect effect, the indirect effect is significant when the 95% confidence interval of sample-based Bootstrap does not contain zero. Results show that the intervening effect of team behavioral integration on the relationship between LMX differentiation and team creativity was −0.72 and the 95% confidence interval of sample-based Bootstrap (20000) was (−1.91, −0.13) (excluded zero). Taken together, Hypothesis 2, team behavioral integration mediates the relationship between LMX differentiation and team creativity, was thus supported.

**FIGURE 2:**
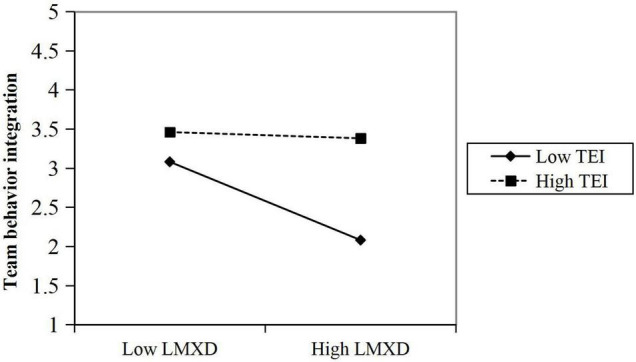
Interaction plot.

H3 predicted that TEI moderates the relationship between LMX differentiation and team behavioral integration. The results in Table 3 showed that the interaction between LMX differentiation and team EI is significantly related to team behavioral integration (M3, β = 0.23, *p* < 0.05). Figure 2 and slope tests demonstrated that the negative relationship between LMX differentiation and team behavioral integration was significantly stronger, when team EI was at low levels (β = −0.24, *p* < 0.01) than at high levels (β = −0.09, ns), the difference is significant (Δ = 0.15, *p* < 0.05). Hypothesis 3 was thus supported.

Finally, we tested H4 which suggested that the mediation effect would be stronger under the low team EI condition. We used the Model 7 of PROCESS (Hayes, 2012) to examine this hypothesis. The results show that the conditional indirect effect of LMX differentiation on team creativity *via* team behavioral integration was non-significant [effect size = −0.18, 95% CI = (−1.10, 0.59)] in the high level of TEI, but the conditional effect was significant [effect size = −1.62, 95% CI = (−3.45, −0.59)] in the low level of TEI. Additionally, there was a significant difference in the estimates of these two mediation effects [Δ = 1.44, 95% CI excluded 0: (1.12, 3.59)]. Thus, Hypothesis 4 was supported.

## Discussion

The goal of this study is to investigate whether, how, and when LMX differentiation influences team creativity. We introduce team behavioral integration and TEI as the mediator and moderator, respectively. Using a survey based on 91 R&D teams from Chinese companies, we confirmed that LMX differentiation is negatively related to team creativity, and team behavioral integration mediates the above relationship. In addition, TEI moderates the indirect relationship between LMX differentiation and team behavioral integration *via* team behavioral integration. Specially, our findings suggest that TEI, as an important process in which team members can adaptively shape and behave differently according to shifting environments (Roberts et al., 2001), has a potential to be an important indicator of the relationship between leader-member exchange and the successful integration of team behavior. The findings point to the importance of studying how LMX differentiation affects team creativity in R&D team settings, especially through the mediating role of team behavioral integration and moderated role of TEI.

### Theoretical Implications

This study contributes to existing team-level research in the following ways.

First, drawing on the IPO model and social comparison theory, this study is among the first to use team behavioral integration as the mechanism to explain the relationship of LMX differentiation and team creativity within organizational environments. Our findings also support prior research that LMX differentiation negatively relates to the team creativity, primarily in R&D teams (Stewart and Johnson, 2009; Liao et al., 2010; Harris et al., 2014). Although other mediators have shown to have an effect on LMX differentiation and team creativity such as relationship conflict (Zhao, 2015), we examined a different mechanism within this relationship. Results found that evidence to further support high LMX differentiation within a team creates a negative context in which team members have the perception of in-group and outgroup differentiation, primarily caused by the perception of relational boundary (Anand et al., 2011), and had injustice perception (Liao et al., 2010). We contribute by offering a better understanding of why and how team behavioral integration can be decreased by negative team climates with high levels of LMX differentiation.

Another finding in this study is that TEI moderated the negative relationship between LMX differentiation and team creativity *via* team behavioral integration. This study expands the proposed mechanisms and also offers new perspective to better understand how emotional intelligence works at the team level. Our findings build on the logic that emotional intelligence is predictive in the teamwork context, particularly in ones with high relational demands, and add more explanation on how TEI helps team members better use and manage emotions, including their own and others. In addition, this study explains how TEI improves team members’ abilities to use and manage emotions effectively in work teams. This processes changes with different leadership treatment styles, more specifically, with high levels of LMX differentiation. Our study also suggests that TEI, as an important process in which team members can adaptively shape and behave differently according to shifting environments (Roberts et al., 2001), can be an important indicator of the relationship between leader-member exchange and the successful integration of team behavior, particularly in the context with high LMX differentiation.

Last, the findings establish TEI’s role in facilitating team creativity. Much of current scholarship has examined emotional expression as important for the overall work experience, and it has been found to be positively related to job satisfaction, job behavior, and job performance. Our findings build upon this notion further, highlighting that TEI contributes to job performance. TEI can directly improve cooperation (Yang and Mossholder, 2004) and information elaboration through its effects on cognition, namely the creative process.

### Practical Implications

There are several practical contributions to this study at the team level. First, for team building, beyond using emotional intelligence as a selection tool to choose team members, human resource managers can utilize it as a development tool to help foster emotionally effective norms during the team-building process (Elfenbein, 2006; MacCann and Roberts, 2008). It will also be helpful for teams to continue to create positive work environments and organizational climates to increase trust and creativity within teams. Our findings also indicate that emotional intelligence testing may be more important for teams that need to hire more knowledge workers to produce creativity in jobs, since team members in these teams need higher degrees of information elaboration to perform their tasks (MacCann and Roberts, 2008).

Second, for team management, interventions on TEI can be used once a team has already been formed. Emotional interventions can be used in training and development programs, which may increase team members’ emotional competences and skills. These programs teach employees to use emotions effectively in their work and develop more effective norms for emotional behaviors (Elfenbein, 2006). Relevant training and development programs, or coaching interventions, can be offered within organizations or by external firms. Program components include practicing mindfulness, journaling, 360° assessment, and leader gap analysis, and actively seeking direct feedback from others (Hopkins and Yonker, 2015). These coaching behaviors and interventions strengthen team members’ ability to reflect on their own behaviors to be more self-aware (Hopkins and Yonker, 2015). In addition, laboratory research and initial field studies reaffirm that cognitive reappraisal interventions can also be effective in altering emotional experiences (Thory, 2013). Thus, emotional regulation strategies such as situation selection and cognitive reappraisal are recommended in teams to increase team members’ emotional intelligence and to help them use and manage emotions more effectively (Parke et al., 2015).

Last, we encourage team leaders, particularly in R&D teams, to continue to invest in building high-quality relationships with their followers. Leaders who engage in LMX differentiation should carefully consider how they develop relationships with followers, as team members with high LMX may be more likely to play informal leadership roles in teams (Boies and Howell, 2006). On the one hand, informal leaders have positive influences on members and have the potential to facilitate effective coordination in teams. On the other hand, team members may perceive the unfairness as well, reducing the relational quality and team potential (Henderson et al., 2009). Therefore, team leaders should also put more effort on building high-quality relationships with followers, because it will be helpful for team members to engage in more creative, vigilant, and responsive processes while at work. Training and development programs should focus on these areas and build other interpersonal skills to facilitate deeper informal and formal mentorship relationships with followers. As aforementioned, 360° leader feedback is recommended in this capacity to help team leaders and followers develop self-awareness and perspective-taking.

### Limitations and Future Research Directions

This study has some noteworthy limitations. First, we limited our analysis to R&D teams from organizations located in Shanghai and Hubei in China and in sectors such as machinery, electronic communication, high-speed railway, and aerospace. The limits the generalizability of the findings to teams in other countries and virtual teams, which might differ in the configuration. Future research can also rectify these limitations by seeking multiple countries’ data and also data from virtual teams. Further, even though the aggregation results of ICC (2) supported, additional complex multilevel analysis (i.e., multilevel latent covariate approach) proposed by Lüdtke et al. (2008) is supposed to use given that aggregation may lead to measurement error if the team is small. Second, we focus on multiple industries without accounting for industry-specific effects on teams’ activities. Given that multiindustry focus means that industry-specific factors such as market demand and market competitiveness which impinge on teams’ activities, future studies could focus on a single sector to see whether the findings would hold. Another potential limitation is that this study only considered the degree of variation in team members’ LMX quality (variation). As such, there are several other methods to measure the LMX variation from multisource indirectly, besides the coefficient of variation that was used in this study (Han and Bai, 2014). Moreover, besides variation in relationship quality between leader-follower dyads in the same work group (i.e., LMX differentiation), recent advances in LMX theory have showed that there may also be inconsistent and conflicting thoughts about the relationship within leader-follower dyads, that is, LMX ambivalence (Lee et al., 2019). Future research can further test its effect on creativity. Other properties and relevant measurements should be considered in future research to enrich the understanding of other patterns of differentiation process.

In addition, one future research direction is to consider educational level as one possible moderator in the theoretical model and explore educational effect more prominently given that the correlation results showed that it has correlated with team creativity (β = 0.26, *p* < 0.05). Another future research direction could continue to focus on team integration concepts outside of behavioral integration, such as affective integration (i.e., how teammates perceive the quality of their interpersonal relationships within the team) and cognitive integration (i.e., the amount teammates comprehend each other’s interpretive frameworks; Cronin et al., 2011). Understanding how effective and cognitive integration mediates the relationships between LMX differentiation and team creativity can help expand upon the benefits of different team-focused integration concepts.

## Conclusion

This study examined the relationship between LMX differentiation and team creativity. Drawing on social comparison theory, we conducted a survey on how LMX differentiation influences team creativity. Our findings highlighted that LMX differentiation is negatively related to team creativity, team behavioral integration mediates the relationship between LMX differentiation and team creativity, and TEI moderates the relationship between LMX differentiation and team behavioral integration. This effect then moderates the indirect relationship of LMX differentiation and team creativity *via* team behavioral integration. These results collectively enhance understanding of how team members interact with each other in the context of LMX differentiation and provide relevant suggestions for organizational team building, management, and development. Team development is an important aspect of organizational effectiveness and performance and should continue to be a priority for human resources managers, particularly in R&D teams.

## Data Availability Statement

The raw data supporting the conclusions of this article will be made available by the authors, without undue reservation.

## Ethics Statement

Ethical review and approval was not required for the study on human participants in accordance with the local legislation and institutional requirements. Written informed consent for participation was not required for this study in accordance with the national legislation and the institutional requirements.

## Author Contributions

JD was responsible for idea generation, manuscript writing for theoretical part, and data collection. XL was responsible for manuscript writing for theoretical part and data analysis. YC was responsible for idea generation and manuscript revision. FS was responsible for data analysis. JA-A was responsible for manuscript revision. All authors contributed to the article and approved the submitted version.

## Conflict of Interest

The authors declare that the research was conducted in the absence of any commercial or financial relationships that could be construed as a potential conflict of interest.

## Publisher’s Note

All claims expressed in this article are solely those of the authors and do not necessarily represent those of their affiliated organizations, or those of the publisher, the editors and the reviewers. Any product that may be evaluated in this article, or claim that may be made by its manufacturer, is not guaranteed or endorsed by the publisher.
